# pCORE, a set of open modular plasmids compatible with golden gate assembly

**DOI:** 10.1038/s41598-026-66483-4

**Published:** 2026-08-15

**Authors:** Louisa Gonzalez Somermeyer, Maxim M. Perfilov, Feng Gao, Dmitry A. Gorbachev, Nikita V. Sytov, Victoria Kovalenko, Macià Sureda-Vives, Aubin Fleiss, Pilar Baldominos, Ilia V. Yampolsky, Fyodor A. Kondrashov, Alexander S. Mishin, Karen S. Sarkisyan

**Affiliations:** 1 Institute of Science and Technology Austria, Klosterneuburg, Austria; 2 Evolutionary and Synthetic Biology Unit, Okinawa Institute of Science and Technology Graduate University, Okinawa, Japan; 3 Shemyakin-Ovchinnikov Institute of Bioorganic Chemistry, Russian Academy of Sciences, Moscow, Russia; 4 Cloning Facility, Moscow, Russia; 5 MRC Laboratory of Medical Sciences, London, UK; 6 Institute of Clinical Sciences, Faculty of Medicine, Imperial College London, London, UK

**Keywords:** Plasmid, Vector, Genetic engineering, Modular assembly, Golden gate assembly, Molecular cloning, Biotechnology, Microbiology

## Abstract

Modular DNA assembly based Golden Gate cloning (MoClo) is a widely used framework to assemble genetic constructs. A number of MoClo “destination vectors” have been developed, however, available options are still limited with regards to copy number and antibiotic resistance in bacteria, vector compactness, and industry researchers’ access to materials. Here, we assembled combinations of five resistance cassettes, four origins of replication and two colour markers to develop a collection of compact MoClo-compatible pCORE destination vectors. pCORE vectors support MoClo Level 1 and Level 2 assembly, and can serve as starting points for developing vectors for other hosts. We tested the performance of our vector collection in *Escherichia coli*, *Agrobacterium tumefaciens* and *Saccharomyces cerevisiae*. pCORE vectors are available to academia and industry under the terms of OpenMTA licence.

**Supplementary Information:**

The online version contains supplementary material available at https://doi.org/10.1038/s41598-026-66483-4.

 Golden Gate cloning relies on restriction endonucleases that cut outside of their recognition sequences^1^. This feature gives users control over nucleotide overhangs post-digestion, allows the recognition site to be removed after ligation of a target molecule, and simplifies assembly of multiple inserts in a single one-pot reaction^2^.

Modular assembly of DNA via Golden Gate cloning has become a popular approach used in synthetic biology for generation of complex genetic constructs. It employs standardised, hierarchical assembly, typically generating transcriptional units from collections of promoters, terminators, tags, and coding regions. These transcriptional units can then be combined to assemble larger constructs. The modular nature and standardised format of DNA fragments enables the reuse and sharing of DNA parts, and continuous integration of new parts into the existing framework. Consequently, modular cloning toolboxes are available for various species, from bacteria^3,4^ to yeast^5,6^, mammals^7^, and plants^2,8,9^. Unfortunately, due to parallel development, assembly syntax and overhang standards differ across the toolboxes.

In our labs, we adapted the MoClo standard proposed by Weber et al.^2^. However, the found limited choice of MoClo destination vectors to support stable co-existence of plasmids in *E.coli* while offering a choice of copy number and antibiotic resistances. To establish standardised molecular infrastructure for our projects, we aimed to assemble a set of core vectors that would provide such options, while also being suitable as a basis for further development of other, host-specific vectors. The resulting pCORE collection of compact MoClo-compatible plasmids represents combinations of five antibiotic resistance cassettes, four origins of replication for *E.coli*, and two colour markers (Fig. 1a).

The pCORE vectors complement MoClo Toolkit vectors (Addgene Kit #1000000044) and support the same overhang standard. However, pCORE vectors are not designed for hierarchical assembly. They are intended for end-use in bacteria, or as a basis for generating new vectors for other host organisms. Instead of supporting hierarchical assembly, pCORE vectors function as dead-end Level 1 or Level 2 vectors, accepting inserts from Level 0 and Level 1 MoClo Toolkit vectors via BsaI or BpiI digestion, respectively (Fig. 1b). They also serve as backbones for creating new specialised vectors by replacement of the entire insert via AarI-based Golden Gate assembly.

**Fig. 1:**
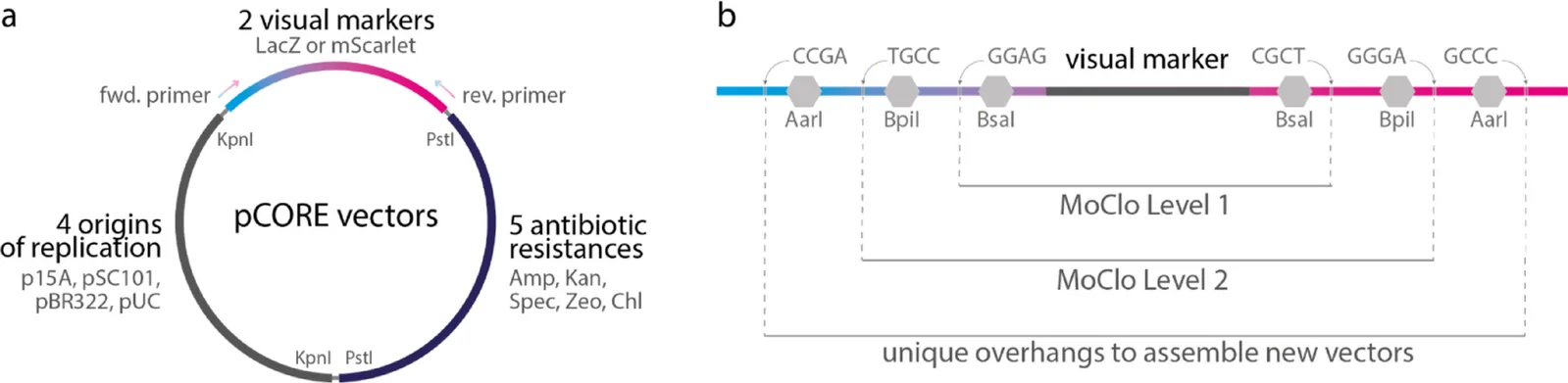
Schematic representation of pCORE vector structure. (a) A standard pCORE vector is formed by three variable parts: one of four origins of replication, one of five antibiotic resistance cassettes and one of two cassettes with marker of colour selection. Flanking sequences for simplified PCR amplification are marked as arrows with labels. (b) Sequences of interest could be cloned into a pCORE vector by GoldenGate reaction using BsaI (standard MoClo Level 1 assembly), BpiI (standard MoClo Level 2 assembly) or AarI.

To construct pCORE vectors, we selected five different antibiotic resistances (spectinomycin, ampicillin, kanamycin, chloramphenicol, and zeocin), four bacterial origins of replication (pUC, pBR322, p15A, and pSC101), and two colour markers (LacZ for blue-white screening, and mScarlet for fluorescence-based screening). We aimed to generate 40 destination vectors representing all combinations of these parts (Table 1). Each vector in the set was assigned a short number-based name for tube-labelling (e.g. pCORE_01), and a more detailed full name describing its components (e.g. pCORE_01__pUC_AmpR_LacZ).

We domesticated all DNA parts to remove recognition sites for the following type IIS restriction enzymes: BsaI, BpiI, BsmbI, SapI and AarI, as well as for SpeI and 8-base-cutters NotI and SbfI. Resistance cassettes were designed to support selection in a wide range of industrially relevant microbes. Expression of resistance genes was designed to be controlled by the broad host promoter pBS3, active in gram-negative and gram-positive bacteria and yeast^10^. Accordingly, a tandem of bacterial terminator Lambda T0 and yeast terminator tAdh1 followed each resistance gene, with the exception of AmpR cassette, where we omitted tAdh1 because ampicillin is not effective against yeast.

To facilitate potential future replacement of resistance cassettes and origins of replication, we introduced additional restriction sites at the ends of the corresponding DNA parts: PstI for freeing resistance cassettes, and KpnI for replication origins. We also inserted constant sequences AATCGGTCACTGTGGCACGTCACCTCGACGCTG and GTGCGGCAGGATGCACTCTTTGAAAATGTACGCC flanking the insert directly at its 5’ and 3’ ends to simplify Sanger sequencing and PCR amplification.

The four origins of replication were selected to have different copy number in *E. coli*, ranging from high (pUC, expected over 500 copies per cell) to medium (pBR322, expected ~ 20 copies per cell), to low (p15A and pSC101, expected 5–10 copies per cell). Importantly, they also belong to different incompatibility groups, with pUC and pBR322 in group A, and p15A and pSC101 in groups B and C, respectively^11–13^. Plasmids from the same incompatibility group cannot stably co-exist due to competition for replication machinery and partitioning control systems. This could lead to improper copy number regulation, and eventually, the loss of all but one plasmid over generations. pCORE vectors from different incompatibility groups can be co-transformed, maintaining their copy number and offering choice of selectable markers.

Of the 40 intended vectors, only 27 were assembled in *E.coli* and verified with Nanopore sequencing (summary available at Supplementary Table 1). This indicates that certain combinations of origins of replication and antibiotic resistance cassettes did not replicate well in *E.coli*, possibly reflecting suboptimal design of some of our transcription units. Most failed assemblies were p15A-based, though some involved pSC101 or pUC origins of replication. All pBR322-based vectors were successfully assembled. Full-plasmid Nanopore sequencing revealed small deviations from the expected sequences, including single-nucleotide polymorphism and small spontaneous deletions or insertions which did not seem to affect the function.

**Fig. 2:**
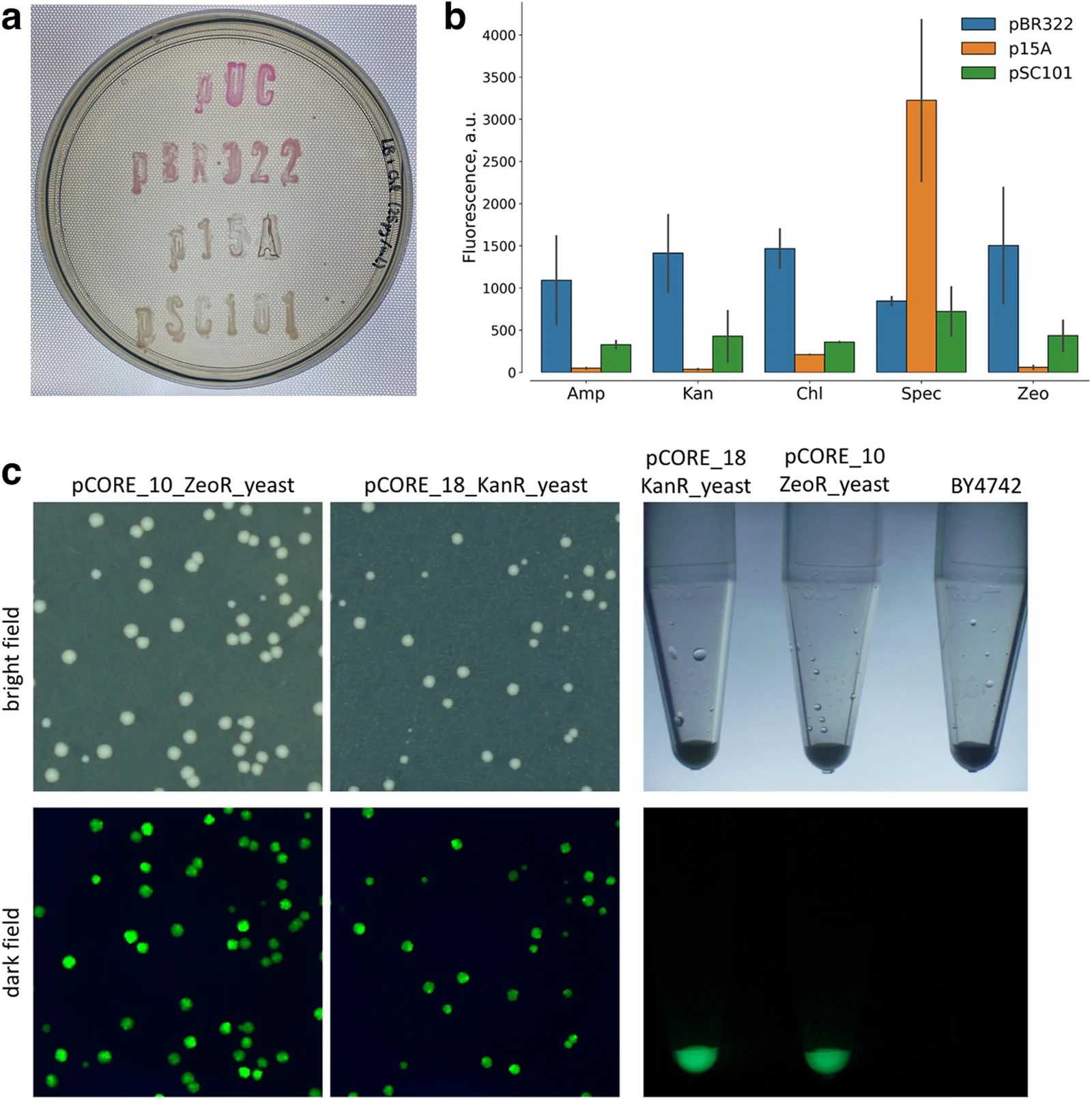
Functional validation of pCORE vectors. **a**. A representative photo of bacterial streaks encoding mScarlet-expressing chloramphenicol-resistant pCORE vectors with different origins of replication. **b**. Intensity of fluorescence of mScarlet-expressing pCORE-vectors in *E.coli*, based on 10,000 FACS events Mean fluorescence is displayed, with error bars representing standard deviation. **с**. Fluorescence imaging of yeast colonies encoding pCORE-derived episomal plasmids with kanamycin and zeocin resistance.

To illustrate the intended use of pCORE vectors, we provide some use-cases below (see also: Fig. 1b):

pCORE as Level 1. pCORE vectors allow BsaI-based assembly of transcription units into any pCORE vector from existing MoClo Level 0 vectors or PCR fragments. This is useful if the original Level 1 MoClo Toolkit vector (large, AmpR, high-copy, T-DNA-containing) or CIDAR Toolkit vector (KanR, high-copy) is unsuitable for the experiment.

pCORE as Level 2. pCORE vectors allow assembly of multi-gene circuits from existing MoClo Level 1 constructs, offering an alternative to the original Level 2 MoClo Toolkit vector (KanR, high-copy, T-DNA-containing). As BpiI sites are located externally to the BsaI sites, BpiI digestion eliminates BsaI sites from the plasmid, allowing the introduction of intermediate BsaI- or BsmBI-containing end-linkers for extended cloning rounds.

pCORE as backbones for new vectors. pCORE vectors facilitate the assembly of new vectors in an AarI-mediated Golden Gate reaction. AarI digestion eliminates BpiI and BsaI sites, enabling introduction of BsaI-, BpiI or BsmBI-containing sequences of custom design. For example, such an assembly may be used to adapt pCORE vectors for use in other hosts by introducing origins of replication, homology arms or additional antibiotic resistance cassettes.

To functionally test the vectors, we grew mScarlet-containing vectors on plates and also analysed them by flow cytometry. As expected, plasmids based on origins of replication with higher copy numbers produced higher levels of mScarlet (Fig. 2ab, Supplementary File 3), with one notable expectation of pCORE_29, a p15-based spectinomycin-resistant vector, showing unexpectedly high levels of fluorescence.

We also tested the functionality of the selectable markers and suitability of the vectors as backbones for other hosts using the yeast *Saccharomyces cerevisiae*. We subcloned 2µ yeast origin of replication and a GFP expression cassette into pCORE_10__pUC_ZeoR_mScarlet and pCORE_18__pBR322_KanR_mScarlet vectors. Transformation of newly cloned plasmids into *S. cerevisiae* resulted in green fluorescent yeast colonies stably growing on plates with corresponding antibiotics (Fig. 2c), illustrating the utility of pCORE vectors as a basis for engineering vectors for new hosts.

Additionally, we tested and expanded the bacterial functionality and usability of the vectors as backbones in the important plant pathogen *Agrobacterium tumefaciens*. We subcloned the agrobacterial pRK2 origin of replication and an mNeonGreen expression cassette driven by the J23116 or J23117 promoters into the pCORE_11__pBR322_AmpR_LacZ vector. Transformation of the newly constructed plasmids into *A. tumefaciens* resulted in different levels of green fluorescent signal (Fig. 3a).

Then, we used the pCORE_11-based vector for visualization of *Agrobacterium* virulence proteins VirB2 and VirB5 involved in plant infection. We expressed Vir-proteins as fusions with bright green fluorescent protein mBaoJin^14^ or with the fluorogen-activating protein DiB3/F53L^15^. Live-cell fluorescence localization microscopy further demonstrated the utility of pCORE vectors in *Agrobacterium* cells (Fig. 3b–d). We also used the mBaoJin-VirB5-encoding vector to monitor the fluorescence signal at several time points during the early stages of *Agrobacterium* infection (Supplementary Fig. 1). The designed agrobacterial pCORE vectors lack left border (LB) and right border (RB) sequences, enabling targeted protein expression exclusively in agrobacterial cells, in contrast to standard MoClo backbones. However, by using LB and RB as modular parts, the agrobacterial pCORE vectors can be readily extended to support independent expression in both agrobacterial and plant cells.

**Fig. 3:**
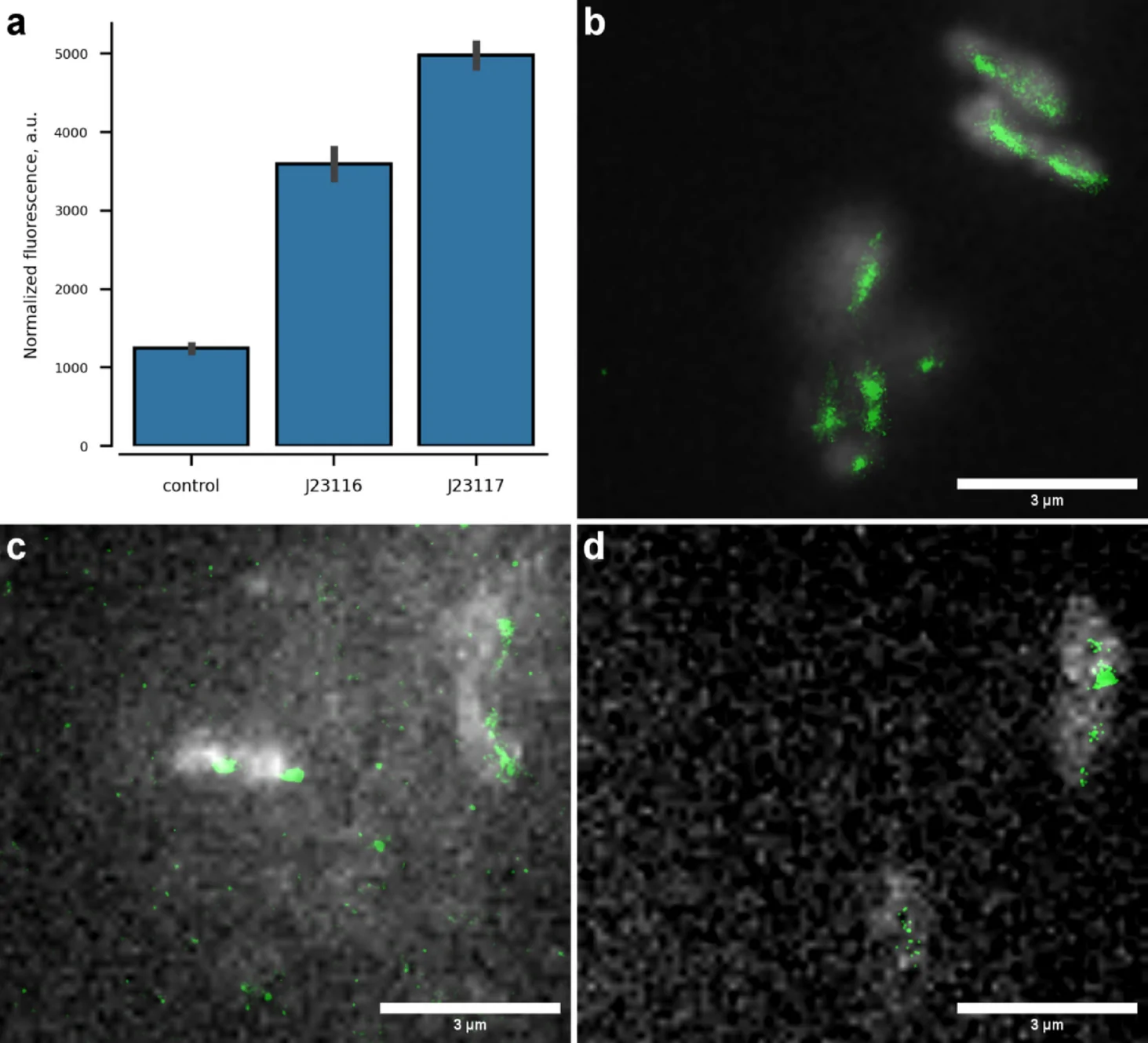
Functional validation of pCORE vectors in *Agrobacterium tumefaciens*. **a**. Intensity of fluorescence of mNeonGreen-expressing pCORE-vectors in *A.tumefaciens*. Mean fluorescence is displayed, with error bars representing standard deviation. b-d.Fluorescence localization microscopy of *Agrobacterium* with pCORE-vectors expressing mBaoJin-VirB5 (**b**), DiB3/F53L-VirB2 (**c**) and DiB3/F53L-VirB5 (**d**). Images are a merge of widefield microscopy (grey) and reconstruction of single-molecule localization microscopy (green).

The twenty seven pCORE vectors reported here, which represent 15 out of 20 initially planned combinations of origins of replication and antibiotic resistances, can be used as backbones for DNA assembly within the MoClo framework, or as a foundation for development of other vectors. The principle of modularity is widely used in modern cloning frameworks, including MoClo^8^, GoldenBraid^16^, *E.coli-*oriented CIDAR^4^, PhytoBricks^17^, uLoop^18^, and others. The flexibility in choice of origins of replication or antibiotic resistance is typically not supported in these kits, with the notable exception of JUMP plasmids^19^ based on Standard European Vector Architecture (SEVA)^20^. pCORE design was developed in parallel to SEVA and shares conceptual similarities with JUMP plasmids. With the exception of Level 1 assembly, pCORE and JUMP are not cross-compatible.

We hope that the pCORE vectors presented here will be useful for the generation of plasmids for experiments in *E.coli*, yeasts and *A.tumefaciens*, and will contribute to open bioeconomy. The usability in other hosts should be tested separately. The plasmids will be made easily available through Addgene to researchers in academia and industry under the terms of OpenMTA^21^.

## Materials and methods

### Vector design

Synthetic DNA parts corresponding to bacterial replication origins (pUC, pBR322, p15A, pSC101), antibiotic resistance cassettes (kanamycin, ampicillin, spectinomycin, chloramphenicol, zeocin), and colour selection marker cassettes (mScarlet or LacZ under pLac promoter and lambda T0 terminator control) were ordered from Twist Bioscience as linear dsDNA. Each part was flanked by restriction sites for SapI leaving specific compatible overhangs (ACC-GTA for replication origins, GTA-AAT for resistance cassettes, AAT-ACC for colour markers), such that SapI sites would be destroyed in the final assembled vectors.

### Plasmid construction

dsDNA parts were combined into full plasmids in single-tube digestion/ligation reactions containing 100 ng of each part, 20U of SapI (NEB), and 10U of T4 ligase in 1X T4 buffer (Thermofisher) in a final volume of 20 µl. Reaction conditions were 25 cycles of 90 s at 37 °C / 180 s at 16 °C, followed by 5 min at 50 °C and 10 min at 80 °C^4^. 10 µl of reaction was used to transform chemically competent *E. coli* cells (Invitrogen) according to the supplier’s protocol. Cells were plated on LB agar containing the appropriate antibiotics, with or without 40 µg/ml X-Gal (Thermofisher) for colour selection via LacZ or mScarlet, respectively. Sequences of individual clones were confirmed via Sanger sequencing (Microsynth) and Nanopore.

### Fluorescence imaging of *Escherichia coli*

*Escherichia coli* cultures transformed with pCORE plasmids with mScarlet selection under pLac promoter control were grown on agar plates containing the appropriate antibiotics at 37 °C for 16 h. Fluorescent bacteria were then imaged in Fusion Pulse (Vilber) or by fluorescence-activated cell sorting (FACS) on FACS Aria III cell sorter (BD Biosciences) (excitation at 561 nm and detection at 670/14 nm). Processing of photos was performed using FiJi ImageJ distribution (version 1.53t)^22^.

### Assembly of yeast vectors using pCORE backbones

The genetic components for *S. cerevisiae* were amplified from the Yeast Toolkit^6^ by PCR using the Encyclo polymerase (Evrogen) and primers that introduced type II restriction sites and overhangs compatible with the chosen acceptor vector (Tables 2 and 3). The GFP expression cassette was obtained as follows. The yeast promoter pRPL18B was amplified from the plasmid pYTK017 using primer_011 and primer_012. The coding sequence of sGFP was amplified from pYTK047 using primer_013 and primer_014. The terminator tPGK1 was amplified from pYTK054 using primer_015 and primer_016. All amplicons were gel-purified using the QIAquick Gel Extraction Kit and assembled into the MoClo Level 1, position 1 acceptor pICH47732. The 2µ origin was amplified from the plasmid pYTK082 using primer_009 and primer_010, gel purified and cloned into the MoClo Level 1, position 2 acceptor pICH47742. The GFP cassette, 2µ origin, and the end linker from pICH41744 were subsequently cloned into pCORE_10 and pCORE_18. Those backbones were selected due to their pUC origin ensuring a high copy number in *E. coli* and the presence of KanR and ZeoR cassettes, which confer resistance to G418 and Zeocin in *S. cerevisiae*.

### Transformation of *S.cerevisiae* and fluorescence imaging

The yeast vectors pCORE_10 and pCORE_18 containing the 2µ origin and sGFP cassette were chemically transformed into *S.cerevisiae* cells using the method described in the ref^23^: per transformation, 10^8^ cells in exponential phase were washed twice in 1 ml of double distilled water, then washed twice in 1 ml of lithium acetate mix (lithium acetate 0.1 M, TE) and cells were recovered in 25 µL of lithium acetate mix. To this mix were added 50 µg of denatured salmon sperm DNA (Invitrogen), 500 ng of plasmid and 300 µL of lithium acetate/PEG solution (lithium acetate 0.1 M, PEG4000 45%, TE). The mix was vortexed for 1 min before incubating the cells for 25 min at 42 °C. The cells were then centrifuged for 2 min at 9000 rpm, resuspended in 1 ml of YPD and left at 30 °C without shaking for 1 h for recovery. The cells were then plated on YPD to check for viability and on YDP supplemented with either G418 (200 µg/ml) or Zeocin (250 µg/ml). Plates were incubated overnight at 30 °C before imaging the clones. Single colonies were imaged using the SINGER PIXL system. To capture bright field images, the White Lighting Mode was used with an exposure time of 5.5 ms. For green fluorescence imaging, the Lighting Mode was switched to Blue (465–485 nm), with the addition of a 527 nm filter and an extended exposure time of 150 ms. Overnight cultures were prepared from single colony inoculations, and the OD was adjusted to 4 for pCORE_10, pCORE_18, and the negative control BY4742. Yeast cultures were centrifuged at 5000 rpm for 2 min to harvest the yeast pellets. Imaging of these pellets was performed using an Olympus SZX16 stereo microscope system and the imaging software cellSens.

### Assembly of *A. tumefaciens* vectors using pCORE backbones

The transcription unit, encoding mNeonGreen under control of J23119 promoter and L3S2P21 terminator flanked with BsmBI restriction sites, was amplified from the P0466 plasmid (constructed in our lab earlier) with primer_021 and primer_022 and cloned into pCORE_11 vector with GoldenGate reaction as described in Plasmid construction with 2U of AarI (Thermo Scientific) restriction enzyme. The resulting plasmid was named pCORE_11 with a modified insert.

The broad-host range origin of replication pRK2 was amplified as two fragments (trfA and oriV) from the MoClo Level 1, position 1 acceptor pICH47732 with primers primer_017 and primer_018, and primer_019 and primer_020. Both fragments were cloned into MoClo Level-1 acceptor pAGM1311 and then assembled together in the MoClo Level 0 acceptor pICH41258. Also the insert with LacZ cassette was amplified from pCORE_11 with primer_023 and primer_024 and then cloned into the same MoClo Level 0 acceptor. Both pRK2 origin and insert sequence were flanked with BsmBI restriction sites that were used to clone these fragments into pCORE_11 vector with modified insert by GoldenGate reaction as described in Plasmid construction. The resulting plasmid was named pCORE_11_pRK2.

The coding sequences of mNeonGreen, VirB2 and VirB5, the synthetic bacterial terminator L3S2P21 and synthetic bacterial promoters J23116, J23117 and J23119 were synthesized by Twist Bioscience (USA). All parts were cloned into corresponding MoClo level 0 acceptor plasmids. MoClo Level 0 plasmids encoding DiB3/F53L and mBaoJin were constructed in our lab earlier. Using pCORE_11_pRK2 as acceptor the plasmids with several transcription units for expression in *A. tumefaciens* were assembled with GoldenGate reactions as described in Plasmid construction: J23116 - mNeonGreen - L3S2P21, J23117 - mNeonGreen - L3S2P21, J23119 - mBaoJin - VirB5 - L3S2P21, J23119 - DiB3/F53L - VirB5 - L3S2P21 and J23119 - DiB3/F53L - VirB2 - L3S2P21.

### Transformation of *A. tumefaciens*

Vectors pCORE_11_pRK2 with transcription units for expression in agrobacterial cells, were transformed into *Agrobacterium tumefaciens* strain AGL0 by electroporation using a preinstalled *Agrobacterium*-specific program on a MicroPulser Electroporator (Bio-Rad Laboratories). Transformed agrobacteria were grown for three days on LB agar plates containing 50 mg/L rifampicin and 200 mg/L carbenicillin at 28 °C. Individual colonies were then inoculated into 10 ml of LB medium containing the same antibiotics. After overnight incubation at 28 °C with shaking at 220 rpm, cultures were centrifuged at 2900 × g, resuspended in 25% glycerol, and stored as glycerol stocks at − 80 °C.

### Fluorescence imaging of *A. tumefaciens*

Glycerol stocks of *Agrobacterium* were inoculated into 10 ml of LB medium containing 50 mg/L rifampicin and 200 mg/L carbenicillin and grown overnight in the dark at 28 °C with shaking at 220 rpm. Cultures were then centrifuged at 2900 × g and resuspended in MMA buffer (10 mM MES [Formedium], 10 mM MgCl₂ [Molecula], and 200 µM acetosyringone [Merck]).

Aliquots of 200 µL of the resuspended *Agrobacterium* cultures were dispensed into µ-Plate 96-well square plates (ibidi GmbH) and placed in a Tecan microplate reader (Tecan Spark). Fluorescence was measured using 485/20 nm excitation and 535/20 nm emission filters. A gain of 131, 30 flashes, and an integration time of 40 µs were used for fluorescence detection. Optical density at 600 nm (OD₆₀₀) was measured for each well and used to normalize the fluorescence signals.

For fluorescence localization microscopy, a 30 µL of *Agrobacterium* cells suspension was diluted in MMA buffer to a final OD₆₀₀ of 0.2, then it was applied on a coverslip for imaging. For imaging *Agrobacterium* cells expressing the fluorogen-activating protein DiB3/F53L, the M739 chromophore was added to a final concentration of 30 nM. Images were acquired with Nanoimager S (ONI) equipped with UPlanSApo 100 × 1.4NA oil immersion lens, 488 nm laser, 560 nm dichroic mirror, and Scope8 sCMOS camera. Images were captured in the total internal reflection fluorescence (TIRF) regime using a 488 nm laser (≈ 475 W/cm^2^). A series of multiple images (1000 frames) were obtained at 20 fps. Image acquisition and reconstruction was performed using NimOS 1.18.3.15066.

For widefield fluorescence microscopy, *Agrobacterium tumefaciens* was added to *Nicotiana tabacum* BY-2 cell cultures to a final OD₆₀₀ of 0.2. The infected cultures were incubated at room temperature, and aliquots were collected at 0.25, 1, 2, and 3 h post-infection. The samples were applied to coverslips and immobilized in 0.5% low-melting-point agarose. Images were acquired with Leica DMI6000b inverted microscope (Leica Microsystem, Germany) equipped with HC PL Apo 100 × 1.40–0.70 oil lens, GFP filter cube (excitation filter 470/40, emission filter 525/50) (Leica Microsystem, Germany), CoolLED pE-300_white_ (CoolLED Ltd, UK) illumination source and Andor Zyla 5.5 CL 10 Tap sCMOS camera (Andor Technology, UK), and using Micromanager software^24^. Image acquisition was performed using the blue channel of CoolLED_white_ pE-300 with illumination intensity of about 0.5–1.5 mW.

**Table 1:** Maps of the assembled and tested vectors as benchling links, with short and long names of plasmids.

Short name	Long name	Map
A summary of all presented pCORE vectors: https://airtable.com/appi45sIkqpSiX5HQ/shrWbqfvdlA95FtFe		
pCORE_02	pCORE_02__pUC_ChlR_LacZ	https://benchling.com/s/seq-rtlhq7JMpCsGKtLwt7vC?m=slm-kbynphHfVzat5mc1zs2m
pCORE_04	pCORE_04__pUC_SpecR_LacZ	https://benchling.com/s/seq-ND9ktAiUiYaNUhNazi9f?m=slm-jZB9824KuNX8ZkERXXOZ
pCORE_05	pCORE_05__pUC_ZeoR_LacZ	https://benchling.com/s/seq-q52vX1boTwpuWTMYUynI?m=slm-OO0TJd8mhST13Wl8Z0ob
pCORE_07	pCORE_07__pUC_ChlR_mScarlet	https://benchling.com/s/seq-FH1UjoCY4FGpHu6PhOJo?m=slm-7fMSmrOnmDDHokTUrkTf
pCORE_10	pCORE_10__pUC_ZeoR_mScarlet	https://benchling.com/s/seq-wSzboVKJ66RhvRKkCkpD?m=slm-Z7N1rcpEC4KQyOBha0GY
pCORE_11	pCORE_11__pBR322_AmpR_LacZ	https://benchling.com/s/seq-AP3KeVK7WPtEwlcKGoZL?m=slm-6XAUFnGc87I4RTspXAuC
pCORE_12	pCORE_12__pBR322_ChlR_LacZ	https://benchling.com/s/seq-Hs49rp4FK7lhdItjDVjx?m=slm-xjH1ue3N4mJ1FbnpJR0p
pCORE_13	pCORE_13__pBR322_KanR_LacZ	https://benchling.com/s/seq-lIBjo09ovXnI8PPBJG2e?m=slm-2zR1xzKif7Jlq8P9Jw8W
pCORE_14	pCORE_14__pBR322_SpecR_LacZ	https://benchling.com/s/seq-Y8q3dAxFI4EGwgYUl6Lx?m=slm-Ghp7MZEHHa8oAH45sGNe
pCORE_15	pCORE_15__pBR322_ZeoR_LacZ	https://benchling.com/s/seq-kfiemPgqm0GJ3VHJepBH?m=slm-UO3k4u7s8P9D1p5s8oIT
pCORE_16	pCORE_16__pBR322_AmpR_mScarlet	https://benchling.com/s/seq-n7xO5mr7eD4bKbBkYpDz?m=slm-oCNowTXZQKbdhVefghxx
pCORE_17	pCORE_17__pBR322_ChlR_mScarlet	https://benchling.com/s/seq-88xdGTtDxzeZIrxJCwvj?m=slm-7yGTwpRypet4LA7IeJop
pCORE_18	pCORE_18__pBR322_KanR_mScarlet	https://benchling.com/s/seq-lOpW6TeVPb2lkXIPd3eC?m=slm-qeauEe65WEQ3DKdOQAfU
pCORE_19	pCORE_19__pBR322_SpecR_mScarlet	https://benchling.com/s/seq-KByvaj4CmdvLo5nFlSae?m=slm-UnWai0SSMpZ0y7kPv35D
pCORE_20	pCORE_20__pBR322_ZeoR_mScarlet	https://benchling.com/s/seq-278V1Gynzuu9O7rYVxbi?m=slm-7SMuHdBumQxOddv24dk3
pCORE_21	pCORE_21__p15A_AmpR_LacZ	https://benchling.com/s/seq-70lY49ieplS5PjhI8cDC?m=slm-CSdnHOaFbAD2LSoTs6Kb
pCORE_24	pCORE_24__p15A_SpecR_LacZ	https://benchling.com/s/seq-58zMDiajLVTNmTNKHKCC?m=slm-TlB3MVx6zo4Ggc5a5atW
pCORE_29	pCORE_29__p15A_SpecR_mScarlet	https://benchling.com/s/seq-LVppQUESR432BGf2dGVA?m=slm-j1S7l6FsrMrvB4fViZsA
pCORE_31	pCORE_31__pSC101_AmpR_LacZ	https://benchling.com/s/seq-51Inb4aTuh4Xu7TLdl6M?m=slm-r9gZoyIikywmgZ1awNWS
pCORE_32	pCORE_32__pSC101_ChlR_LacZ	https://benchling.com/s/seq-StcEBzVMC2LEW651cy4M?m=slm-BnphOBfxQPU0A3SzYSGw
pCORE_33	pCORE_33__pSC101_KanR_LacZ	https://benchling.com/s/seq-gxHHunSru9K8hTPI7okh?m=slm-t45IJIPXKscTmNmuUdjX
pCORE_34	pCORE_34__pSC101_SpecR_LacZ	https://benchling.com/s/seq-R7NjPO0jXt3m4JLKm1f6?m=slm-SMLgle6CK8Rs8UCVNSCH
pCORE_36	pCORE_36__pSC101_AmpR_mScarlet	https://benchling.com/s/seq-JC2JgkktLJweLoF4kZ3A?m=slm-rfWkmSZleF4WEGBLXKxx
pCORE_37	pCORE_37__pSC101_ChlR_mScarlet	https://benchling.com/s/seq-58yIreVmrDfFc9RHchTo?m=slm-d6Ik5JEpMs4LYpfOskWm
pCORE_38	pCORE_38__pSC101_KanR_mScarlet	https://benchling.com/s/seq-sNTR80o40FmMZ18OoWYu?m=slm-1k1JxxRR0AQRFVNlPOZJ
pCORE_39	pCORE_39__pSC101_SpecR_mScarlet	https://benchling.com/s/seq-ANAAzS3TWduEmljAk7if?m=slm-4aROol9GP9pWJPuGM9AN
pCORE_40	pCORE_40__pSC101_ZeoR_mScarlet	https://benchling.com/s/seq-wMtwSzZd8HQmtK3Bjn3X?m=slm-oV1HiJkHY3sCI5GEr6tX

**Table 2:** Primers used in this study.

Primer name	Sequence 5’ -> 3’	Purpose
primer_011	tagtcGGTCTCaGGAGcagaggatgtccaatattttttttaagg	Forward PCR primer for promoter from yeast tool kit
primer_012	aatctGGTCTCaACCTgatcttttgttttttgttttcttc	Reverse PCR primer for promoter from yeast tool kit
primer_013	acgttGGTCTCaAGGTaGAGACGatgcgtaaaggcgaagagctg	Forward for PCR of sfGFP coding region from yeast kit
primer_014	aagcgGGTCTCtAAGCtGAGACGtcatttgtacagttcatccatacc	014 @R for PCR of sfGFP coding region from yeast kit
primer_015	tagtcggtctcaGCTTtaactcgagattgaattg	015 @F for PCR of terminator from yeast kit
primer_016	ggCctggtctccagcGcatagaaatatcgaatggg	016 @R for PCR of terminator from yeast kit
primer_009	tagtcCgtctcaGGAGaacgaagcatctgtgcttcatt	009 @F for PCR of 2micron ori from yeast kit
primer_010	agtctCgtctcaAGCggaagttcctattctctag	010 @R for PCR of 2micron ori from yeast kit
primer_017	aattGGTCTCaacataatgCGTCTCaCCGAatatGCGGCCGCGTGAGCGACAAAAGCTTTAAtgtggcgcaacgatgcc	017 @F for PCR of pRK2 part1 ori
primer_018	tgagGGTCTCAACAAcgctggctgctgaaccc	018 @R for PCR of pRK2 part1 ori
primer_019	aattGGTCTCaacatagcgccgggctggttgccc	019 @F for PCR of pRK2 part2 ori
primer_020	tgagGGTCTCAACAAACCTCGTCTCaAGTAatatGCGGCCGCTTATGGGCGCGTATTTCCCAccgggagggttcgagaagg	020 @R for PCR of pRK2 part2 ori
primer_021	cactgattagCACCTGCTGCCccgaCgagacgTTGACAGCTAGCTCAGT	021 @F for PCR of mNeonGreen transcription unit
primer_022	gatagcaactCACCTGCTGCCgggcGgagacgGGACCAAAACGAAAAAA	022 @R for PCR of mNeonGreen transcription unit
primer_023	ctgtggtctcaaatgcgtctcaAGGAgctgcctgtatcgagtggtgccgagattgcaggtgcgc	023 @F for PCR of LacZ insert for agrobacterial pCORE
primer_024	tcgtggtctcaacctcgtctcaAAAGcggctggcacatacaaatgggggcccttgcaggtgg	024 @R for PCR of LacZ insert for agrobacterial pCORE

**Table 3:** Plasmids used as templates for PCR.

Plasmid name	Link	Benchling	Purpose
pYTK017	https://www.addgene.org/65124/sequences/	https://benchling.com/s/seq-n00FcYNJROxB7lnAiXw6?m=slm-tWtBrGCRNp1f2TUlYjV4	Template for yeast promoter pRPL18B
pYTK047	https://www.addgene.org/65154/sequences/	https://benchling.com/s/seq-h8umERiFFIHphBUo4XSQ?m=slm-ZfWIk164tpsG2BKddTV2	Template for sGFP coding sequence
pYTK054	https://www.addgene.org/65161/sequences/	https://benchling.com/s/seq-oGQFJFrtPyBxfQwvlQPc?m=slm-zh3lArGaNC4fXu4UyCdM	Template for yeast terminator tPGK1
pICH47732	https://www.addgene.org/48000/sequences/	https://benchling.com/s/seq-6dwIGuFVhIXa5BPpYgIP	MoClo Level 1, position 1 acceptor backbone
pYTK082	https://www.addgene.org/65189/sequences/	https://benchling.com/s/seq-ZWXXHfU4jAgCH8TvAk8h?m=slm-YM3CgYQroHDq2Vd5ZgzR	Template for 2µ yeast replication origin
pICH47742	https://www.addgene.org/48001/sequences/	https://benchling.com/s/seq-aZ0bs42rFg9XyOSoNfwI?m=slm-kh4MRSlivOJ3Tue3GWls	MoClo Level 1, position 2 acceptor backbone
pICH41744	https://www.addgene.org/48017/sequences/	https://benchling.com/s/seq-c9jpVvDrErfWM27shb8Y	Level 2 end linker
P0466		https://benchling.com/s/seq-OIeeyteyyuBsmA8wdJwe?m=slm-tT33WEbx8wMmmUEshPRu	Template for mNeonGreen transcription unit

## Supplementary Information

Below is the link to the electronic supplementary material.

Supplementary Material 1

Supplementary Material 2

Supplementary Material 3

Supplementary Material 4

Supplementary Material 5

## Data Availability

All data generated or analysed during this study are included in this published article and its supplementary information files. The plasmids are available to researchers in academia and industry from Addgene (https://www.addgene.org/Karen_Sarkisyan/) under the terms of OpenMTA. The summary table for all 40 plasmids is available at https://airtable.com/appi45sIkqpSiX5HQ/shrWbqfvdlA95FtFe/tblktOeYTv7a11D2B.

## Abbreviations

AmpR: Ampicillin resistance cassette
FACS: Fluorescence-activated cell sorting
GFP: Green fluorescent protein
KanR: Kanamycin resistance cassette
MoClo: Modular DNA assembly based Golden Gate cloning
OpenMTA: Open Material Transfer Agreement
PCR: Polymerase chain reaction
