# Supplementary material for: pCORE, a set of open modular plasmids compatible with golden gate assembly
Source: Sci Rep. 2026 Aug 15;16:35150. doi: 10.1038/s41598-026-66483-4 (PMC13719502; doi:10.1038/s41598-026-66483-4)
Supplement: Supplementary file 1 [file 41598_2026_66483_MOESM4_ESM.pdf]

Supplementary Table 1. The list of designed pCORE vectors.

Name	Map link	Origin, incompatibility group, number of copies in *E.coli*	Resistance	Marker gene	Successfully constructed and sequenced?
pCORE_01__pUC_AmpR_LacZ	https://benchling.com/s/seq-uzrfFDMiJzBkosv36hce?m=slm-O9pR63DuCoIuo5JlFCnf	pUC | group A | ~500-700 copies (pMB1 derivative)	Ampicillin	LacZ	No
pCORE_02__pUC_ChlR_LacZ	https://benchling.com/s/seq-rtlhq7JMpCsGKtLwt7vC?m=slm-kbynphHfVzat5mc1zs2m	pUC | group A | ~500-700 copies (pMB1 derivative)	Chloramphenicol	LacZ	Yes
pCORE_03__pUC_KanR_LacZ	https://benchling.com/s/seq-7LiJYWwInX0ixjaWex29?m=slm-qzXZuBInawr2yYRuh3kh	pUC | group A | ~500-700 copies (pMB1 derivative)	Kanamycin	LacZ	No
pCORE_04__pUC_SpecR_LacZ	https://benchling.com/s/seq-ND9ktAiUiYaNUhNazi9f?m=slm-jZB9824KuNX8ZkERXXOZ	pUC | group A | ~500-700 copies (pMB1 derivative)	Spectinomycin	LacZ	Yes
pCORE_05__pUC_ZeoR_LacZ	https://benchling.com/s/seq-q52vX1boTwpuWTMYUynI?m=slm-OO0TJd8mhST13Wl8Z0ob	pUC | group A | ~500-700 copies (pMB1 derivative)	Zeocin	LacZ	Yes
pCORE_06__pUC_AmpR_mScarlet	https://benchling.com/s/seq-n9CzfQY1yyiKAhPRCfOD?m=slm-jTvqnY6ZOLusVyE0Oqwm	pUC | group A | ~500-700 copies (pMB1 derivative)	Ampicillin	mScarlet	No
pCORE_07__pUC_ChlR_mScarlet	https://benchling.com/s/seq-FH1UjoCY4FGpHu6PhOJo?m	pUC | group A | ~500-700 copies (pMB1 derivative)	Chloramphenicol	mScarlet	Yes

	=slm-7fMSmrOnmDDHokTUrkTf				
pCORE_08__pUC_KanR_mScarlet	https://benchling.com/s/seq-X5lFj5qlHhs64h7vmVEc?m=slm-j9UQD5s9p2qcYdNduetW	pUC | group A | ~500-700 copies (pMB1 derivative)	Kanamycin	mScarlet	No
pCORE_09__pUC_SpecR_mScarlet	https://benchling.com/s/seq-KQ5W1eTfRK95xykQhBxY?m=slm-6iWDK7sRudl6v2nIg9YB	pUC | group A | ~500-700 copies (pMB1 derivative)	Spectinomycin	mScarlet	No
pCORE_10__pUC_ZeoR_mScarlet	https://benchling.com/s/seq-wSzboVKJ66RhvRKkCkpD?m=slm-Z7N1rcpEC4KQyOBha0GY	pUC | group A | ~500-700 copies (pMB1 derivative)	Zeocin	mScarlet	Yes
pCORE_11__pBR322_AmpR_LacZ	https://benchling.com/s/seq-AP3KeVK7WPtEwlcKGoZL?m=slm-6XAUFnGc87I4RTspXAuC	pBR322 | group A | ~15-20 copies	Ampicillin	LacZ	Yes
pCORE_12__pBR322_ChlR_LacZ	https://benchling.com/s/seq-Hs49rp4FK7lhdItjDVjx?m=slm-xjH1ue3N4mJ1FbnpJR0p	pBR322 | group A | ~15-20 copies	Chloramphenicol	LacZ	Yes
pCORE_13__pBR322_KanR_LacZ	https://benchling.com/s/seq-lIBjo09ovXnI8PPBJG2e?m=slm-2zR1xzKif7Jlq8P9Jw8W	pBR322 | group A | ~15-20 copies	Kanamycin	LacZ	Yes
pCORE_14__pBR322_SpecR_LacZ	https://benchling.com/s/seq-Y8q3dAxFI4EGwgYUl6Lx?m=slm-Ghp7MZEHHa8oAH45sGNe	pBR322 | group A | ~15-20 copies	Spectinomycin	LacZ	Yes

pCORE_15__pBR322_ZeoR_LacZ	https://benchling.com/s/seq-kfiemPgqm0GJ3VHJepBH?m=slm-UO3k4u7s8P9D1p5s8oIT	pBR322 | group A | ~15-20 copies	Zeocin	LacZ	Yes
pCORE_16__pBR322_AmpR_mScarlet	https://benchling.com/s/seq-n7xO5mr7eD4bKbBkYpDz?m=slm-oCNowTXZQKbdhVefghxx	pBR322 | group A | ~15-20 copies	Ampicillin	mScarlet	Yes
pCORE_17__pBR322_ChlR_mScarlet	https://benchling.com/s/seq-88xdGTtDxzeZIrxJCwvj?m=slm-7yGTwpRypet4LA7IeJop	pBR322 | group A | ~15-20 copies	Chloramphenicol	mScarlet	Yes
pCORE_18__pBR322_KanR_mScarlet	https://benchling.com/s/seq-lOpW6TeVPb2lkXIPd3eC?m=slm-qeauEe65WEQ3DKdOQAfU	pBR322 | group A | ~15-20 copies	Kanamycin	mScarlet	Yes
pCORE_19__pBR322_SpecR_mScarlet	https://benchling.com/s/seq-KByvaj4CmdvLo5nFlSae?m=slm-UnWai0SSMpZ0y7kPv35D	pBR322 | group A | ~15-20 copies	Spectinomycin	mScarlet	Yes
pCORE_20__pBR322_ZeoR_mScarlet	https://benchling.com/s/seq-278V1Gynzuu9O7rYVxbi?m=slm-7SMuHdBumQxOddv24dk3	pBR322 | group A | ~15-20 copies	Zeocin	mScarlet	Yes
pCORE_21__p15A_AmpR_LacZ	https://benchling.com/s/seq-70lY49ieplS5PjhI8cDC?m=slm-CSdnHOaFbAD2LSoTs6Kb	p15A | group B | ~10 copies	Ampicillin	LacZ	Yes
pCORE_22__p15A_ChlR_LacZ	https://benchling.com/s/seq-UX62cZklyuosHMXdnlCX?m=slm-oXlZ4XnpsDiixMZe1m2M	p15A | group B | ~10 copies	Chloramphenicol	LacZ	No

pCORE_23__p15A_KanR_LacZ	https://benchling.com/s/seq-OuqyAUU1z0fIiGGAPuLW?m=slm-LMyMPcOnPFpqkbznRhR6	p15A | group B | ~10 copies	Kanamycin	LacZ	No
pCORE_24__p15A_SpecR_LacZ	https://benchling.com/s/seq-58zMDiajLVTNmTNKHKCC?m=slm-TIB3MVx6zo4Ggc5a5atW	p15A | group B | ~10 copies	Spectinomycin	LacZ	Yes
pCORE_25__p15A_ZeoR_LacZ	https://benchling.com/s/seq-ajmx4fFnTQdv5uIaHpr3?m=slm-1bzr8Gy223X6BiLqb6cL	p15A | group B | ~10 copies	Zeocin	LacZ	No
pCORE_26__p15A_AmpR_mScarlet	https://benchling.com/s/seq-9yIhfhJSZc67fndqWcK8?m=slm-ZCFApnAqd9qUwq56OTsM	p15A | group B | ~10 copies	Ampicillin	mScarlet	No
pCORE_27__p15A_ChlR_mScarlet	https://benchling.com/s/seq-yGJTiRruwMHOs5tJQdCV?m=slm-l7XfLlWsHVoJ3hUMNT7X	p15A | group B | ~10 copies	Chloramphenicol	mScarlet	No
pCORE_28__p15A_KanR_mScarlet	https://benchling.com/s/seq-oQbtyKFrAMgfZsqVN086?m=slm-So7u2hDWr01KphzDvOiY	p15A | group B | ~10 copies	Kanamycin	mScarlet	No
pCORE_29__p15A_SpecR_mScarlet	https://benchling.com/s/seq-LVppQUESR432BGf2dGVA?m=slm-j1S7l6FsrMrvB4fViZsA	p15A | group B | ~10 copies	Spectinomycin	mScarlet	Yes
pCORE_30__p15A_ZeoR_mScarlet	https://benchling.com/s/seq-P3k4QTqqqyBAmOz5Dal6?m=slm-UkHih9BvpZy7HlVZdEm	p15A | group B | ~10 copies	Zeocin	mScarlet	No

	4				
pCORE_31__pSC101_AmpR_LacZ	https://benchling.com/s/seq-51Inb4aTuh4Xu7TLdl6M?m=slm-r9gZoyIikywmgZ1awNWS	pSC101 | group C | ~5 copies	Ampicillin	LacZ	Yes
pCORE_32__pSC101_ChlR_LacZ	https://benchling.com/s/seq-StcEBzVMC2LEW651cy4M?m=slm-BnphOBfxQPU0A3SzYSGw	pSC101 | group C | ~5 copies	Chloramphenicol	LacZ	Yes
pCORE_33__pSC101_KanR_LacZ	https://benchling.com/s/seq-gxHHunSru9K8hTPI7okh?m=slm-t45IJIPXKscTmNmuUdjX	pSC101 | group C | ~5 copies	Kanamycin	LacZ	Yes
pCORE_34__pSC101_SpecR_LacZ	https://benchling.com/s/seq-R7NjPO0jXt3m4JLKm1f6?m=slm-SMLgle6CK8Rs8UCVNSCH	pSC101 | group C | ~5 copies	Spectinomycin	LacZ	Yes
pCORE_35__pSC101_ZeoR_LacZ	https://benchling.com/s/seq-aqPs6gJKbQArIWvchsbG?m=slm-olNu50lVEAiTbus9nHM1	pSC101 | group C | ~5 copies	Zeocin	LacZ	No
pCORE_36__pSC101_AmpR_mScarlet	https://benchling.com/s/seq-JC2JgkktLJweLoF4kZ3A?m=slm-rfWkmSZIeF4WEGBLXKxx	pSC101 | group C | ~5 copies	Ampicillin	mScarlet	Yes
pCORE_37__pSC101_ChlR_mScarlet	https://benchling.com/s/seq-58yIreVmrDfFc9RHchTo?m=slm-d6Ik5JEpMs4LYpfOskWm	pSC101 | group C | ~5 copies	Chloramphenicol	mScarlet	Yes
pCORE_38__pSC1	https://benchling.com/s/seq-	pSC101 | group C | ~5 copies	Kanamycin	mScarlet	Yes

01_KanR_mScarlet	sNTR80o40FmMZ18OoWYu?m=slm-1k1JxxRR0AQRFVNlPOZJ				
pCORE_39__pSC101_SpecR_mScarlet	https://benchling.com/s/seq-ANAAzS3TWduEmljAk7if?m=slm-4aROoI9GP9pWJPuGM9AN	pSC101 | group C | ~5 copies	Spectinomycin	mScarlet	Yes
pCORE_40__pSC101_ZeoR_mScarlet	https://benchling.com/s/seq-wMtwSzZd8HQmtK3Bjn3X?m=slm-oV1HiJkHY3sCI5GEr6tX	pSC101 | group C | ~5 copies	Zeocin	mScarlet	Yes

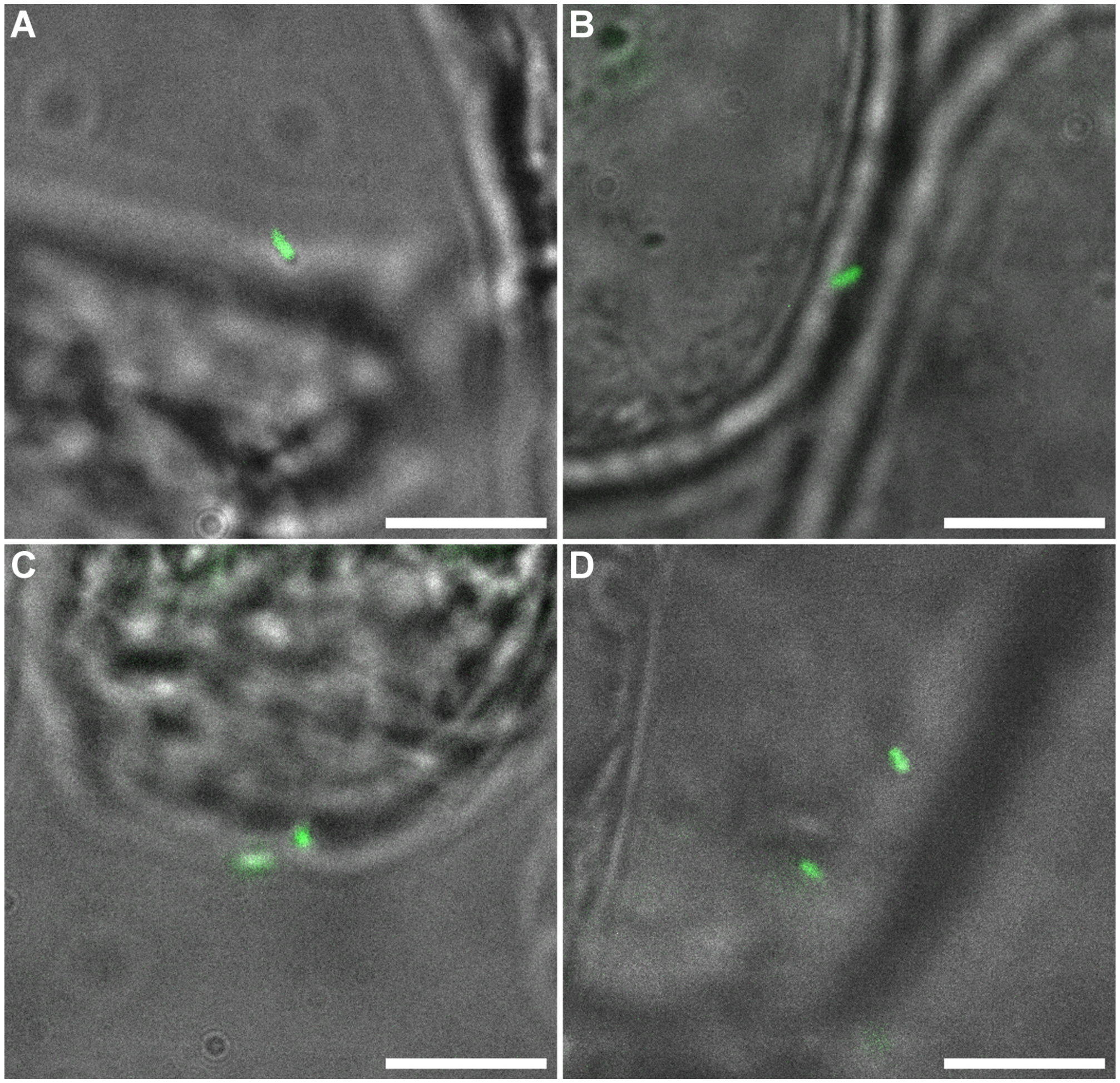

Supplementary Figure 1. Widefield fluorescence microscopy of *Nicotiana tabacum* BY-2 cells infected with *Agrobacterium tumefaciens* carrying a pCORE vector encoding mBaoJin-VirB5. Representative widefield fluorescence microscopy images (A–D) were acquired at 0.25, 1, 2, and 3 h post-infection, respectively. The images are overlays of the transmitted-light channel (gray) and the green fluorescence channel (green). All samples were immobilized in 0.5% low-melting-point agarose. Scale bars, 10 μm.
