# Supplementary material for: pCORE, a set of open modular plasmids compatible with golden gate assembly
Source: Sci Rep. 2026 Aug 15;16:35150. doi: 10.1038/s41598-026-66483-4 (PMC13719502; doi:10.1038/s41598-026-66483-4)
Supplement: Supplementary file 2 [file 41598_2026_66483_MOESM5_ESM.docx]

Supplementary materials

Supplementary File 1. Sequences of all the parts used in this study.

Supplementary File 2. Maps of all vectors as .gb files.

Supplementary File 3. Images of *E.coli* transformed with various pCORE plasmids on agar plates.

Supplementary File 4. Supplementary Table 1 and Supplementary Figure 1.
